# Community strategies for health promotion and prevention of chronic non-communicable diseases with a focus on physical activity and nutrition: the URO/FOCOS study protocol

**DOI:** 10.3389/fpubh.2023.1268322

**Published:** 2024-01-12

**Authors:** Matías Monsalves-Álvarez, María Teresa Solis-Soto, María Soledad Burrone, Alejandro A. Candia, Emilio Jofré-Saldía, Gabriela Espinoza, Marcelo Flores-Opazo, Carlos Puebla, Denisse Valladares-Ide, Sebastián Jannas-Vela

**Affiliations:** Instituto de Ciencias de la Salud, Universidad de O’Higgins, Rancagua, Chile

**Keywords:** healthy lifestyle, community-based participatory research, primary prevention, non-communicable chronic disease, nutritional status, exercise

## Abstract

Non-communicable diseases (NCDs) account for 71% of all annual deaths, totaling 41 million people worldwide. The development and progression of these diseases are highly related to the environment and lifestyle choices, among which physical inactivity and excess malnutrition stand out. Currently, in Chile, there is no evidence at the regional and local level on the impact of physical activity and healthy nutrition plans and interventions on health promotion, prevention, and timely treatment of NCDs. The following protocol delineates the URO/FOCOS (Universidad Regional de O’Higgins/FOrtaleciendo COmunidades Saludables- Regional University of O’Higgins/Strengthening Healthy Communities) study, which will assess pilot community intervention strategies using a participatory action research approach by identifying barriers and facilitators on the practice of physical activity and healthy eating habits. In this project, the community from the O’Higgins region will be involved throughout the entire research process to develop strategies that promote regular physical activity and healthy eating practices. We propose three interrelated strategies: (1) Participatory Action Research, (2) Community interventions for promoting physical activity and healthy nutrition practices, and (3) health education. The URO/FOCOS study offers a unique opportunity in the O’Higgins region to develop participatory strategies and interventions based on the community’s needs and motivations with regard to physical activity and healthy eating habits. We believe these strategies will help to improve the community’s overall health through effective changes in their decision and preferences toward a more active lifestyle and healthier nutrition practices.

## 1 Introduction

Non-communicable diseases (NCDs) account for 71% of all annual deaths, totaling 41 million people worldwide (1). In Chile, the situation is even more severe, with 86% of deaths related to NCDs. This is mainly due to cardiovascular diseases and cancer, which account for 27 and 26% of the all-cause mortality prevalence, respectively (2). NCDs can affect people at any stage of their life cycle (children, adults, and older adults), and although there is a substantial genetic component, the development, and progression of these diseases are highly related to the environment – unhealthy lifestyle habits – among which physical inactivity and excess malnutrition stand out.

Physical inactivity is a major risk factor for the development of NCDs (3), accounting for 11% of healthcare costs worldwide (4) and contributing to 6–10% of the national mortality rate (5). Hence, regular physical activity rises as an effective tool for preventing and reducing NCDs. Unfortunately, in Chile, only 19% of people over 18 years old meet the minimum physical activity recommendations suggested by the World Health Organization (WHO) (6). Furthermore, less than 50% of children and adolescents meet this standard. From a local perspective, the O’Higgins Region (1 of 16 regions from Chile, equating to 4.7% of the country’s population) follows the same trend, being the second most inactive region in the country, and where gender differences and socioeconomic level influence people when it comes to practicing physical activity (6).

Malnutrition or unhealthy diets is another important environment-related risk factor for the development of NCDs. In Chile, according to the Healthy Eating Index, only 5% of the population consumes a healthy diet, and the rest require changes in their eating habits (7). Likewise, only 40% of the children between 6 and 15 in the O’Higgins Region have a normal nutritional status; 27% are overweight, and 28% are obese (8). Importantly, lower-income families and older adults are more prone to nutritional deficiencies, which have significant adverse consequences for physical, cognitive, and general health. Consequently, a high percentage of the regional population presents cardiovascular risk, is overweight, and obese, being one of the regions most affected by NCDs, which translates into increased mental health leave, earlier mortality rates, and lower quality of life (9).

Although several advances are being made in Chile, both in the prevention and control of NCDs, the levels of physical inactivity and eating patterns have not changed substantially in the last two decades (6), most probably because these strategies do not include the local and provincial communities in the design, implementation, and evaluation of these interventions. Moreover, the COVID-19 pandemic has further exacerbated these problems (10, 11), leading to poorer overall health. In this context, it is necessary to develop strategies developed with the local community and key social actors that focus on improving dietary and physical activity patterns and, therefore, people’s health. In this sense, Participatory Action Research (PAR) has been reported as an excellent approach to develop healthy communities. PAR is defined as a “systematic investigation, with the collaboration of those affected by the issue being studied, for education and taking action or effecting social change,” engaging those who live in the community in every phase of the research process (12). Community members bring to the process of knowing and creating knowledge and acting on that knowledge to bring about change (12).

The URO/FOCOS (Universidad Regional de O’Higgins/FOrtaleciendo COmunidades Saludables – Regional University of O’Higgins/Strengthening Healthy Communities) study aims to assess pilot community intervention strategies using a PAR approach by identifying barriers and facilitators on the practice of physical activity and healthy eating habits. These strategies will be designed to have territorial and cultural relevance, promote health, prevent NCDs, and ensure timely treatment within the communities of the O’Higgins Region in Chile.

## 2 Methods

### 2.1 Study design

**Figure 1:**
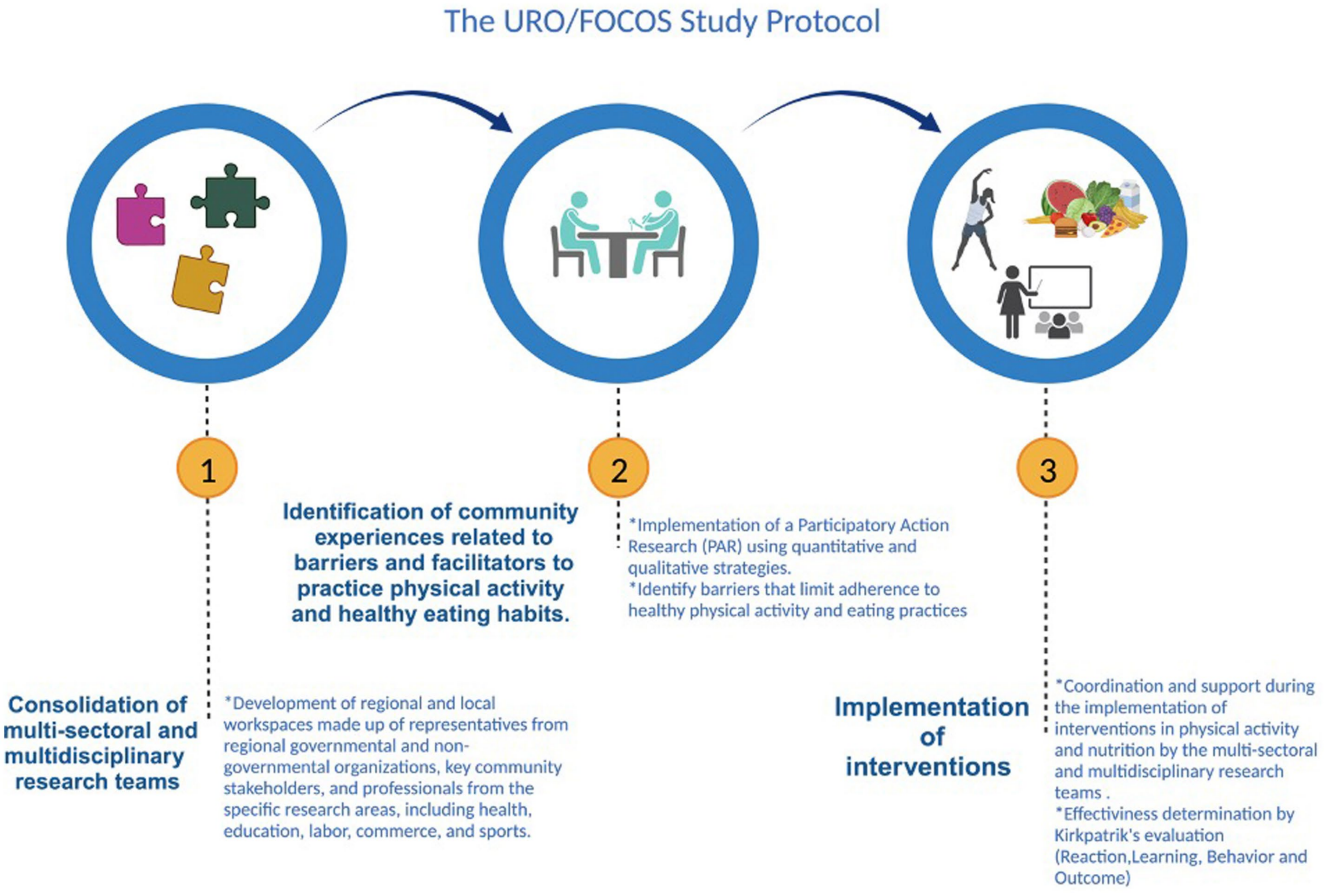
Phases of the project focusing on participatory action research. Created with BioRender.com.

URO/FOCOS project is based on a participatory action-research (PAR) study design, considering 3 phases (Figure 1). The Project will begin in 2023 and will last 2 years.

#### 2.1.1 Phase 1: consolidation of multi-sectoral and multidisciplinary research teams

The consolidation of the multi-sectoral and multidisciplinary research teams will be given by the development of regional and local workspaces comprised of representatives from regional governmental and non-governmental organizations, key community representatives, and professionals from specific research areas, including health, education, labor, commerce, and sports, among others. Likewise, an open call to the community will be implemented, so those members interested in participating, can join the team. These teams will provide their perspectives and visions for decision-making and have a comprehensive understanding of the factors influencing physical activity and nutrition practices. Moreover, they will advise multi-sectoral and multidisciplinary research teams and community members in designing, developing, and evaluating interventions. A socialization meeting with all the parties involved will be organized, where the project’s purpose will be explained and the exchange of ideas will be promoted. The work methodology will also be collectively agreed upon and followed throughout the project.

#### 2.1.2 Phase 2: identification of community experiences related to barriers and facilitators to practice physical activity and healthy eating habits

This phase will be developed using quantitative and qualitative strategies by implementing PAR. Based on the results of this phase, the main problems (e.g., barriers) that limit adherence to healthy physical activity and eating practices will be identified. Facilitators that can be reinforced in the community will also be identified. With this information, evidence-based interventions will be designed, together with the communities, to promote healthy lifestyles in physical activity and nutrition by adapting them to their local context and resources. Based on the national evidence, we will propose to work *a priori* in three areas of interventions:

1. Educational and continuous training strategies: aimed at specific groups (e.g., schoolteachers, primary healthcare professionals, etc.) with a “Train the Trainer” approach to broaden the beneficiary group.
2. Awareness and sensitization strategies: fairs, exhibitions, and other interventions that visualize the problem and the benefits of regular physical activity and healthy eating.
3. Interventions on health prevention and promotion: generate evidence of the effectiveness of specific exercise routines/activities/diets in relation to the promotion and prevention of health problems.

#### 2.1.3 Phase 3: implementation of interventions

Communities will be coordinated and supported during the implementation of the interventions in physical activity and nutrition by the multi-sectoral and multidisciplinary research teams. Local community knowledge, attitudes, and practices will be considered in the design of interventions to promote physical activity and healthy eating. Before and after the interventions, information will be collected to evaluate its effectiveness (details in “*Description of evaluation tools and instruments*”) and assessed through Kirkpatrik’s evaluation model, which considers four different levels: Reaction (Satisfaction), Learning (Acquired knowledge), Behavior, and Outcome (Impact) (13).

In phases 2 and 3, strategies to keep the community informed and achieve community involvement, awreness, and empowerment will be implemented. Thus, in this study, tools will be used that will allow the creation of links of reflection-dialogue-action-learning between the people of the communities involved and external agents consisting of members of the university, students, and members of governmental and non-governmental organizations interested in promoting actions for the development and socio-political empowerment of communities. In this way, the project will emphasize the community’s potential to identify and solve problems. Also, community members will actively promote physical activity and healthy eating habits. The study design is based on learning anchored in the community’s real life and agreements from the position of horizontality between cooperating agents. Thus, the project proposes the co-construction of knowledge, including the contribution from the experience and joint practice between the participants (14).

### 2.2 Description of sites

The O’Higgins Region, located in the Central zone of Chile, is the sixth most populated of the 16 regions and the fourth most densely populated, with 24% of the population living in rural areas. Agriculture, the food industry, and mining stand out among its main economic activities. The region is divided into three provinces: Cardenal Caro, Colchagua, and Cachapoal, with a rural population of 49, 35, and 19%, respectively. The local communities that will be included in the study are Navidad (Cardenal Caro), Placilla (Colchagua), and Rengo (Cachapoal).

Concerning the gender and age distribution, the region has a Masculinity Index of 1.0; 19% are under 15 years of age, and 14% are over 65 years, which reflects a process of aging of the population derived from the advanced demographic transition (15). The O’Higgins Region presents high rates of NCDs, a high prevalence of obesity, and a high rate of physical inactivity. In this regard, it has been observed that 74% of the adult population in Chile and 56% of children between the ages of 6 and 15 suffer from overweight or obesity (8, 9). Furthermore, recent statistics indicate that 93% of children between 11 and 17 years old do not meet the minimum physical activity recommendations, while 76% of adults fail (16).

### 2.3 Participants

A two-stage sampling design will be implemented in the study. First, the multi-sectoral and multidisciplinary research teams will choose the communities in a participatory way, considering one rural and one urban community for each province. The communities will be selected through a purposive non-probability method (17) by assessing the feasibility and geographic access criteria. Subsequently, a specific study population will be chosen in each selected community. As a preliminary idea to be discussed during phases 1 and 2 of the project, it is proposed.

For the qualitative approach, 60–72 people over 18 years will be invited to participate in six focus groups (10–12 people per group, three provinces, and urban and rural communities). We will invite the general population to participate through health services and educational communities, aiming for gender balance. In-deep interviews will also be conducted with key actors identified in each selected community to complement the information explored.

On the other hand, in phases 2 and 3, methods and techniques such as community forums, visioning exercises, and other activities to promote the participation and empowerment of communities, involve representatives, and keep communities informed will be promoted.

This proposal is based on a participatory research approach, attempting to build a theoretical framework to contextualize a “new” scientific approach. It considers relevant the experience of the participants (subjects) as a starting point, an educational process oriented toward group activities that develop a horizontal pedagogical relationship with external agents (researchers, facilitators, etc.), the establishment of ties of learning and reflection, the organization of groups and action, and the active participation of people involved in project activities, such as programming, production of materials and evaluation.

These methodologies will seek to develop a collective process of discussion and reflection, collectivize individual knowledge, enrich it, truly enhance collective knowledge, and create a common educational reflection experience (18). In particular, Community Action Research (CAR) confronts the challenges of producing practical, helpful knowledge to people in everyday conduct, valuing knowing-in-action. The implementation of CAR strategies will be discussed with the research team to foster relationships and collaboration among different actors, creating settings for collective reflection that enable people from various organizations to “see themselves in one another” and establishing cross-institutional links to sustain transformative changes (19).

For the quantitative component, sampling strategies that are representative and feasible to implement in each community (Navidad, Placilla, and Rengo), considering age groups (see Table 2) will be contemplated.

In the implementation phase of the interventions, to promote healthy lifestyles in physical activity and nutrition, efforts will be made to reach approximately 1,000 participants in the three areas of interventions considered *a priori*.

This study received ethics approval from the Comité de Ética Científica (Universidad de O’Higgins, Rancagua, Chile) #050-2022. All participants will sign a written Consent or Assent Form before participating in the study.

### 2.4 Outcomes and other measures

A qualitative and quantitative approach will be used to achieve the objectives of the study’s second phase.

#### 2.4.1 Qualitative approach

This component will identify barriers and facilitators of physical activity and healthy eating habits through interviews with key stakeholders and focus group discussions with local communities. It will provide the vision and position of certain groups on the subject, as well as their lens and perceptions of the practice of physical activity and healthy eating habits. Moreover, dimensions of the health determinant model at the individual level, lifestyles, social and community networks, living and working conditions, and socioeconomic, cultural, and environmental conditions will be explored.

#### 2.4.2 Measurements and procedures

There will be 4 analysis axes where the following dimensions will be studied:

1. Connection with the project
2. Individual
3. Social
4. Environmental

Within these dimensions, the following aspects will be examined: Knowledge, Attitudes, Practices, Barriers, Facilitators, and Recommendations (Table 1).

**Table 1:** Semi-structured interview and focus group guide.

Dimensions	Aspects	Categories
Connection with the project Individual Social Environmental	Knowledge	Physical activity and nutrition
		Benefits
		Programs and recommendations
		Sources of information
	Attitudes	Physical activity and nutrition
	Practices	Physical activity
		Nutrition
		Food selection, purchase, and preparation
		Participation in programs
	Barriers	Physical activity
		Nutrition
	Facilitators	Physical activity and nutrition
		Media
		Programs and national policies
	Recommendations	

#### 2.4.3 Quantitative approach

It will seek to quantify aspects related to knowledge, attitudes, and practices (KAP) concerning physical activity and healthy eating habits. In general, KAP studies explore misconceptions or misunderstandings that may represent obstacles to implementing activities and potential barriers to behavior. In this context, it will:

- Identify specific populations of interest, considering differences between age, gender, and rurality.
- Diagnose the current situation of the target population through standardized and validated instruments and tests.
- Gather information and data on the community’s current programs, focusing on physical activity and nutrition.

#### 2.4.4 Measurements and procedures

A specialized multidisciplinary team of trained researchers will conduct questionnaires and biological and functional tests. In each assessment (interview and measurement), general data, variables regarding health conditions, and biological parameters will be evaluated. Below is a summary table with the measurements in the different populations and phases (Table 2). Subsequently, each of the measures is described.

- Sociodemographic variables: sex, educational level (no studies, incomplete primary education or lower, complete basic education, unfinished high school, incomplete high school, complete high school, incomplete higher technical, complete higher technical, complete and incomplete university and postgraduate), age (years completed), main activity (working, studying, housewife, unemployed, retired or pensioner, other activity), tobacco and alcohol consumption.
- Knowledge and attitudes toward healthy lifestyles (diet and physical exercise): These variables will be explored with instruments agreed upon with the community so that they adapt to the local culture and context (20).
- GHQ-12 Questionnaire: The questionnaire measures mental health. It consists of 12 propositions that must be answered by choosing one of the possible answers given to the participant on a Likert-type scale. It covers four fundamental psychiatric areas: depression, anxiety, social inadequacy, and hypochondria (21).
- SDQ Questionnaire: The Questionnaire is used to measure mental health in children. It detects probable cases of mental and behavioral disorders in children aged 4–13 years. The SDQ consists of 25 items on emotional and behavioral problems experienced by children aged 4–16. It has five subscales: emotional symptoms, conduct problems, hyperactivity/attention problems, peer problems, and social behavior (22).
- Lifestyle habits
- Eating habits: For this, the participant will be asked to report food consumption during a typical week, specifying approximate portions and types of food (9).
- Physical activity habits: The person will be asked to report the activity habits during the last week (7 days), specifying the time and intensity of the physical activity (9). In addition, a subsample of the intervention population will be provided with accelerometers for seven consecutive days, objectively measuring the daily physical activity performed. The results of the questionnaire and the accelerometers will be compared (23).
- Sleep quality habits: Participants will report compliance with hours of sleep (9).
- Body composition: Weight, height, waist and hip circumference, and fat percentage will be measured using a digital scale and stadiometer.
- Cardiovascular markers: A blood, saliva, or hair sample will be taken at the beginning of the interventions and 6 months later. Among the biological markers in the blood to be measured, the following stand out:
- Biochemical profile: Glucose, insulin, cholesterol, triglycerides Got-Ast transaminase, Gpt-Alt transaminase, urea, total proteins, and albumin.
- Vitamin profile: Vitamins A, E, B, C, and D.
- Glycosylated hemoglobin (HbA1c) According to the Clinical Guide for the Management of Type 2 Diabetes, an HbA1c level ≤ 7% is defined as adequate, and an HbA1c level ≥ 7% as inadequate.
- Epigenetic profile, levels of circulating microRNAs in plasma, and gene methylation in immune cells.
- Blood pressure: systolic blood pressure (SBP) and diastolic blood pressure (DBP) will be measured using a digital sphygmomanometer.
- Functional tests: a series of general function tests specific to the populations to be intervened will be performed. The following are the most important:
- 6-min walking test (children, adults, and older adult): a fast walking test that measures the distance to be walked in 6 min (24–26).
- Hand grip strength: the participant must squeeze a static element with your hand, and it will measure the force you exerts on it (27, 28).
- Leg power test: the participant must perform a downward movement (squat) and immediately perform an upward movement that produces a jump. In addition, you must descend to a half squat position, hold this position for approximately 3 s, and then jump (29).
- Arm flexion: the participant must flex and extend the elbows starting from a prone position with the only support of the toes and hands (Children and adults).
- Horizontal jump: with feet together, the participant should jump forward as far as possible (30, 31).
- Short Physical Performance Battery (SPPB): three specific physical performance activities: balance, walking speed in 4 m, and strength through the test of getting up and sitting down from the chair (32).

**Table 2:** Assessment tools used for each age group.

Age group	Assessment tools
Children (6–17 y)	Legal guardians Sociodemographic variables Mental Health (SDQ questionnaire) Healthy life habits Children Body composition Healthy life habits Cardiovascular markers Functional tests
Adults (18–64 y)	Sociodemographic variables Mental Health (GHQ-12 questionnaire) Healthy life habits Body composition Cardiovascular markers Functional tests
Older adults (≥65 y)	Sociodemographic variables Mental Health (GHQ-12 questionnaire) Healthy life habits Body composition Cardiovascular markers Functional tests

GHQ-12, General Health Questionnaire 12; SDQ, Strength and Difficulties Questionnaire.

Specific strategies according to the characteristics of the communities and the particular groups to intervene will be designed. For these interventions, the research group will identify study variables to be assessed before and after the intervention. The selection of variables will be defined according to the specific objective of each intervention. *A priori*, each variable in the same person will be measured at least twice (before and after).

### 2.5 Data analysis

For the qualitative approach, the interviews and focus group discussions will be audio recorded and transcribed, eliminating any identification of the person interviewed. Constant Comparative Analysis, proposed by Glaser and Strauss, will be developed for data analysis (33). First, we will encode the units of meaning (words, sentences, and paragraphs). Subsequently, the codes will be integrated into categories based on their common properties; overlapping categories are integrated (sub-categories are generated), establishing categorization and developing theoretical concepts from the data. For this analysis, we will use Atlas. Ti software.

The quantitative information will be recorded in an online questionnaire and then exported to the statistical program SPSS for further analysis. The absolute and relative frequency will be recorded for the categorical variables, and for the quantitative variables, the central tendency and dispersion measures will be reported. The Chi-square or T-test will be used to analyze differences between two groups, depending on the behavior of the variables. Subsequently, multivariate regression models will be computed to identify lifestyle predictive factors. These predictive factors will be adjusted for potential confounding variables (e.g., age, sex, educational level).

### 2.6 Ethical considerations

Before beginning each interview, the participants — and in the case of minors, their parents, or caregivers – will undergo an informed consent process approved by the Ethical Committee of Human Subjects Research of the Universidad de O’Higgins #050-2022. The participants will not receive any payment or reward for participating in the study, and any incurred costs (transportation, beverage) will be covered by the project or reimbursed to ensure that their participation did not represent a financial burden.

## 3 Discussion

In the URO/FOCOS project, the community from the O’Higgins region will be involved throughout the entire research process to develop strategies that promote regular physical activity and healthy eating. To address this, we propose three interrelated approaches:

Participatory Action Research (PAR): This will include the active work of various regional actors and members of multi-sectoral and multidisciplinary research teams, with whom a participatory investigative process will be developed to diagnose and evaluate the current situation (physical/physiological attributes of the population), as well as barriers and facilitators regarding the practice of physical activity and healthy nutrition in local communities.

Community interventions for promoting physical activity and healthy nutrition practices: This strategy seeks to work with the local communities in designing, implementing, and evaluating the interventions on physical activity and nutrition.

Education: The evaluation of training needs in the local communities is projected in this strategy based on the information provided from the PAR process to provide updated evidence-based training on physical activity and nutrition for health, healthy lifestyle habits, and implementation of physical activity programs for the population, among others.

Some limitations could arise during the implementation of the study. For instance, the access to the research setting, which includes the full participation of the research team, avoiding the influence of potential conflicts of interest, and understanding and implementing real collaborative work. Also, the limitations inherent during the implementation of the quantitative and qualitative study components (e.g., internal and external validity) and the adequate interpretation of the results. Due to the characteristics of the study, it is possible that not all the results can be generalized; however, the method can be extrapolated to other contexts. On the other hand, aligning timeframes in a dynamic context must be considered, which will imply working on flexible planning but considering the administrative deadlines for the execution of the project.

The URO/FOCOS project offers a unique and innovative opportunity in the O’Higgins region to develop participatory strategies that promote regular physical activity and healthy eating based on the community’s needs and motivations. We hope these strategies improve the community’s overall health and, in the long term, create healthy physical activity and nutrition habits, establishing a precedent for effective lifestyle interventions in Chile.

## Author contributions

MM-Á: Writing – original draft, Writing – review & editing. MS-S: Conceptualization, Writing – review & editing. MB: Conceptualization, Writing – review & editing. AC: Writing – review & editing. EJ-S: Writing – review & editing. GE: Writing – review & editing. MF-O: Writing – review & editing. CP: Writing – review & editing. DV-I: Conceptualization, Writing – review & editing. SJ-V: Conceptualization, Writing – review & editing, Funding acquisition, Writing – original draft.
